# Acute Median Neuropathy Due to Persistent Median Artery Thrombosis Following Cupping Therapy: The Diagnostic Value of High-Resolution Ultrasound

**DOI:** 10.7759/cureus.115331

**Published:** 2026-08-27

**Authors:** Wei-Ting Wu, Yu-Chun Hsu, Ke-Vin Chang, Levent Özçakar

**Affiliations:** 1 Physical Medicine and Rehabilitation, National Taiwan University Hospital Bei-Hu Branch, Taipei, TWN; 2 Physical Medicine and Rehabilitation, Department of Physical Medicine and Rehabilitation and Community and Geriatric Research Center, National Taiwan University Hospital Bei-Hu Branch, Taipei, TWN; 3 Department of Physical and Rehabilitation Medicine, Hacettepe University Medical School, Ankara, TUR

**Keywords:** arterial thrombosis, carpal tunnel syndrome, doppler ultrasonography, median neuropathy, persistent median artery, ultrasonography

## Abstract

Persistent median artery (PMA) thrombosis is a rare cause of acute median neuropathy that may mimic typical carpal tunnel syndrome. We report the case of a 45-year-old man with several months of intermittent numbness without weakness affecting the radial three-and-a-half digits of the right hand who developed rapidly worsening pain, forearm erythema and swelling, distal coldness, and finger weakness following acupuncture and cupping therapy. High-resolution ultrasonography revealed a bifid median nerve and an enlarged PMA containing a long-segment hypoechoic thrombus. Spectral Doppler demonstrated progressive flow impairment, with preserved proximal pulsatile flow progressing to a dampened monophasic waveform at the proximal carpus and then to absent flow distal to the carpal tunnel inlet. Preserved flow in the ulnar and common digital arteries indicated adequate collateral perfusion. Anticoagulation with enoxaparin followed by dabigatran etexilate resulted in substantial pain relief. This report demonstrates the value of high-resolution gray-scale and Doppler ultrasonography for identifying PMA thrombosis, characterizing arterial occlusion, and assessing associated median nerve and distal vascular involvement. PMA thrombosis should be considered in rapidly progressive median neuropathy accompanied by severe pain, distal coldness, or other signs of vascular compromise.

## Introduction

Persistent median artery (PMA) is an embryonic vascular remnant that may persist into adulthood and accompany the median nerve through the forearm and carpal tunnel [1]. Although usually asymptomatic, its close anatomical relationship with the median nerve can become clinically relevant when pathological changes such as enlargement, aneurysm formation, or thrombosis develop [2]. In particular, PMA thrombosis is a rare but important cause of acute or rapidly progressive median neuropathy, as enlargement of the thrombosed vessel may compress the adjacent median nerve, while arterial occlusion may simultaneously compromise neural and distal hand perfusion.

The resulting presentation may resemble conventional carpal tunnel syndrome, making early recognition challenging. However, severe or rapidly progressive pain, distal coldness, swelling, or other signs of vascular compromise should prompt consideration of an underlying vascular abnormality. High-resolution ultrasonography is particularly useful in this setting because it allows simultaneous assessment of the median nerve, PMA, intraluminal thrombus, and associated hemodynamic changes without exposure to ionizing radiation. Doppler ultrasonography can further characterize the extent of arterial occlusion and evaluate distal and collateral perfusion, which may have important implications for treatment planning. We report a case of PMA thrombosis associated with a bifid median nerve in a patient with pre-existing intermittent median-distribution numbness who subsequently developed rapidly progressive median neuropathy and vascular manifestations temporally associated with acupuncture and cupping therapy. This report highlights the diagnostic value of multimodal ultrasonography in recognizing vascular causes of atypical median neuropathy.

## Case presentation

A 45-year-old man, weighing 82 kg and measuring 171 cm in height, with a long history of manual labor involving repetitive heavy lifting, had experienced intermittent numbness and tingling involving the radial three-and-a-half digits of his dominant right hand for several months, without associated weakness. He was a nonsmoker and had no known underlying medical or cardiovascular disease. Because these intermittent symptoms were initially considered musculoskeletal in origin, he underwent acupuncture at the Neiguan (PC6) acupoint, followed by cupping therapy over the volar wrist and distal forearm. Over the following days, his clinical condition deteriorated abruptly. He developed diffuse erythema and swelling of the distal forearm, severe wrist pain associated with marked coldness of the hand, and progressive weakness of finger flexion. The inflammatory appearance initially suggested cellulitis superimposed on carpal tunnel syndrome.

**Figure 1:**
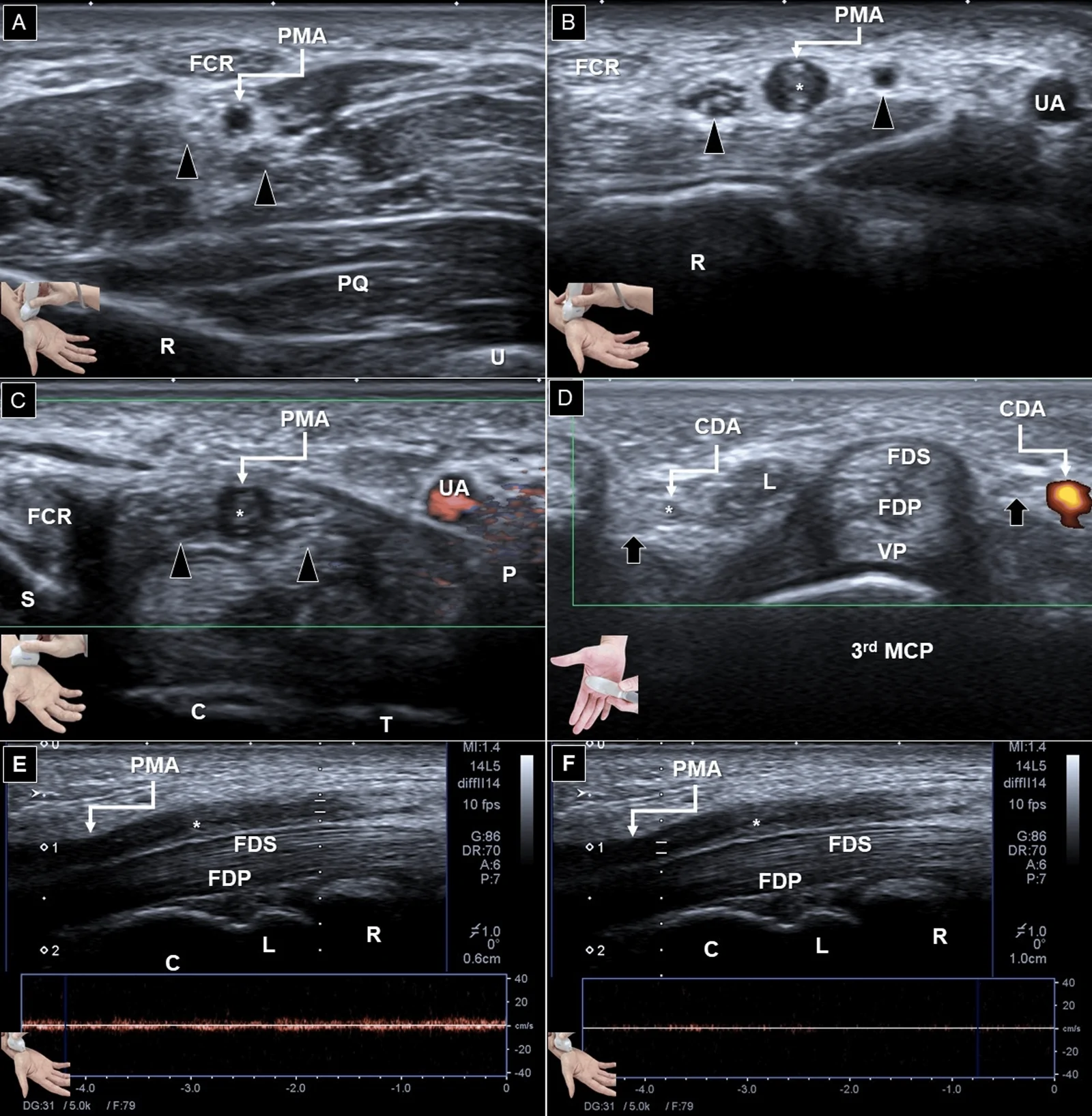
Ultrasonography of PMA thrombosis Short-axis ultrasound image showing a patent PMA between the two divisions of the bifid median nerve proximal to the thrombus (A). An enlarged PMA containing a hypoechoic intraluminal thrombus was identified from the palmar aspect of the distal radioulnar joint (B), through the carpal tunnel (C), and distally to the level of the common digital artery (D). The absence of intraluminal flow on Doppler imaging supported the presence of long-segment PMA thrombosis. Spectral Doppler demonstrated a low-velocity, dampened monophasic waveform at the proximal carpus (E), whereas no detectable arterial waveform was identified distal to the carpal tunnel (F), supporting complete distal occlusion of the PMA. Asterisk: thrombus; black arrowhead: bifid median nerve; black arrow: common digital nerve R: radius; U: ulna; L: lunate; C: capitate; UA: ulnar artery; MN: median nerve; PMA: persistent median artery; FDS: flexor digitorum superficialis; FDP: flexor digitorum profundus; PQ: pronator quadratus; FCR: flexor carpi radialis; CDA: common digital artery; VP: volar plate

Physical examination demonstrated tenderness over the volar wrist, decreased sensation within the median nerve distribution, and a noticeably colder hand compared with the contralateral side, raising suspicion of concomitant vascular compromise. Musculoskeletal ultrasound examination was conducted using an Aplio 300 ultrasound system equipped with a linear transducer (14L5, 5-14 MHz; Canon Medical Systems Corporation, Otawara, Japan). Gray-scale ultrasonography demonstrated an enlarged PMA coursing between the two divisions of the bifid median nerve (Figure 1A), with a long-segment hypoechoic intraluminal thrombus extending from the distal radioulnar joint (Figure 1B), through the carpal tunnel (Figure 1C), and to the metacarpal level (Figure 1D).

The adjacent median nerve appeared mildly swollen and hypoechoic (Figure 1C), suggesting neuropathic involvement potentially related to both mechanical compression by the thrombosed PMA and compromised neural perfusion. No evidence of flexor tenosynovitis, ganglion cyst, abscess formation, or significant soft-tissue edema was observed. Doppler imaging demonstrated complete absence of flow distal to the thrombus, whereas preserved flow was observed in the adjacent ulnar artery (Figure 1C) and the common digital artery between the third and fourth digits (Figure 1D), indicating maintained collateral perfusion despite complete occlusion of the persistent median artery. Spectral Doppler demonstrated a preserved pulsatile waveform proximal to the thrombus, which progressively transitioned to a low-velocity, dampened monophasic waveform at the proximal carpus (Figure 1E). No detectable arterial waveform was identified distal to the carpal tunnel inlet (Figure 1F), supporting complete distal occlusion of the persistent median artery.

The patient was immediately referred to the cardiology department and started on subcutaneous enoxaparin (60 mg twice daily). His pain and forearm swelling improved substantially within the first week, with the visual analog scale score decreasing from 10 to 3. Anticoagulation was subsequently transitioned to oral dabigatran etexilate (110 mg twice daily). During regular cardiology follow-up over more than three months, no residual symptoms were observed.

## Discussion

The PMA represents the persistence of the embryonic axial artery supplying the developing upper limb. Normally, the median artery regresses around the eighth week of gestation as the radial and ulnar arteries mature [3]. Failure of involution results in a persistent vessel that may remain closely associated with the median nerve throughout the forearm. Two anatomical patterns have been described: an antebrachial type that terminates within the forearm and a palmar type that traverses the carpal tunnel and extends into the hand [1]. The latter is of greater clinical relevance because of its close relationship with the median nerve and its potential contribution to the arterial supply of the hand [4]. The PMA may arise from the anterior or common interosseous artery, although origins from the ulnar, radial, or brachial arteries have also been reported [5].

Distally, the PMA often coexists with a bifid median nerve, with a reported prevalence ranging from 9% to 19% [4,6]. The presence of a PMA within the carpal tunnel may contribute to mechanical compression of the median nerve, potentially compromising its blood supply and resulting in signs and symptoms of carpal tunnel syndrome [7]. Given the tightly confined anatomy of the carpal tunnel [8], a large-caliber PMA or pathological changes involving the artery, such as aneurysm, atherosclerosis, calcification, or thrombosis, may further increase local pressure and exacerbate median nerve compression [9].

**Figure 2:**
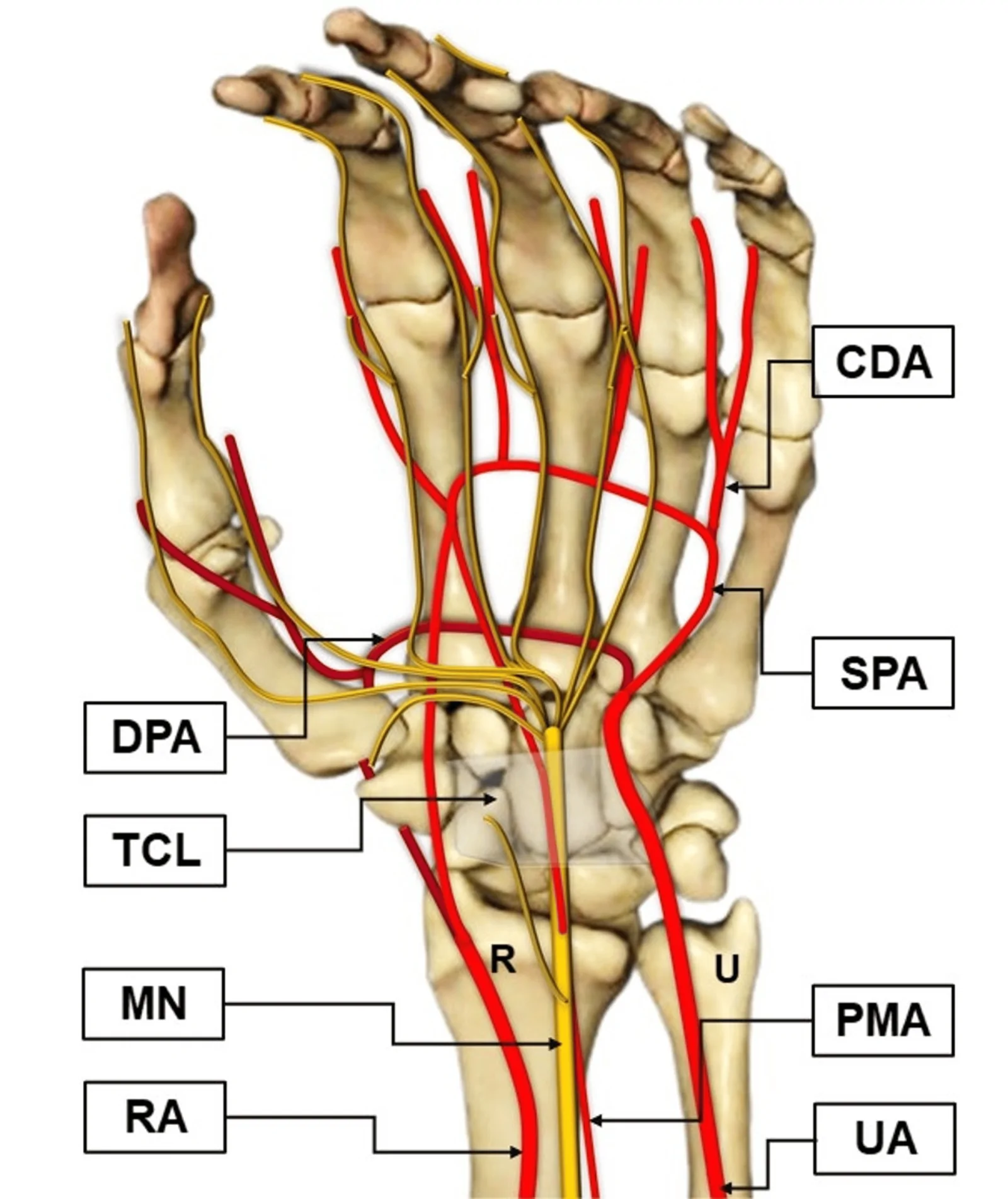
Illustration of the course of PMA Illustration of the course of the PMA and its anatomical relationship with the median nerve, providing a clear overview of the arterial course observed in this case R: radius; U: ulna; RA: radial artery; UA: ulnar artery; DPA: deep palmar arch; SPA: superficial palmar arch; MN: median nerve; PMA: persistent median artery; TCL: transverse carpal ligament; CDA: common digital artery This illustration was manually created by the author (Wei-Ting Wu) using Microsoft PowerPoint (Microsoft Corporation, Redmond, WA)

The PMA also contributes to the vascular supply of the median nerve, with both structures traversing the carpal tunnel (Figure 2) [1]. It may contribute to the superficial palmar circulation by anastomosing with the ulnar artery to form the superficial palmar arch or may terminate as common digital arteries supplying the index and middle fingers (Figure 2) [10]. This variable arterial anatomy has important implications when PMA thrombosis occurs. Enlargement of the thrombosed vessel within the confined carpal tunnel can compress the adjacent median nerve, while arterial occlusion may simultaneously compromise distal perfusion [11]. Nevertheless, complete PMA thrombosis does not necessarily result in critical digital ischemia because perfusion may be maintained through the radial and ulnar arteries and their communications within the palmar arterial arches [7]. The clinical consequences therefore depend on the contribution of the PMA to the digital circulation and the adequacy of collateral perfusion.

PMA thrombosis has been associated with repetitive or strenuous wrist activity, blunt trauma, vibration, local infection, and other conditions predisposing to vascular injury or thrombosis [2,12]. Previous reports have suggested that repetitive mechanical stress may precipitate thrombosis in an otherwise asymptomatic vessel [13]. In the present case, the patient had a long history of repetitive heavy manual work and had experienced intermittent numbness in the median nerve distribution for several months before the acute event, suggesting that an underlying anatomical predisposition or pre-existing median nerve involvement may already have been present. He was a nonsmoker and had no known underlying medical or cardiovascular disease. Nevertheless, an unrecognized systemic thrombotic predisposition cannot be completely excluded based on the available clinical information.

Although local mechanical stimulation has been proposed as a potential precipitating factor for thrombosis in susceptible vessels, the relative contributions of the pre-existing PMA anatomy, repetitive occupational loading, and the subsequent procedures cannot be determined in this patient. Accordingly, acupuncture and cupping therapy should be regarded as temporally associated events rather than established causes of the PMA thrombosis. Clinically, the distinction between the pre-existing and acute phases was notable. Before the procedures, the patient experienced intermittent numbness without weakness. In contrast, the subsequent development of severe pain, forearm erythema and swelling, marked distal coldness, and progressive finger weakness indicated a new neurological and vascular process that could not be explained by carpal tunnel syndrome alone. This combination of neuropathic, inflammatory-appearing, and ischemic manifestations prompted vascular evaluation and ultimately led to the diagnosis of PMA thrombosis.

High-resolution ultrasonography was particularly valuable because neural [14] and vascular abnormalities [15] could be evaluated during the same examination [16]. Gray-scale imaging delineated the bifid median nerve and the enlarged PMA and directly demonstrated the long-segment intraluminal thrombus, while Doppler examination characterized its hemodynamic consequences. In addition to establishing the initial diagnosis, ultrasonography may also provide a convenient means of monitoring thrombus resolution and documenting restoration of arterial flow after treatment. In our patient, Doppler flow remained detectable in the ulnar artery and the common digital artery between the third and fourth digits, indicating preserved collateral perfusion despite PMA occlusion. These findings illustrate how substantial vascular occlusion may coexist with distal coldness and pain without the immediate development of overt digital ischemia or necrosis, owing to preserved collateral perfusion.

Management of PMA thrombosis should therefore be individualized according to the severity of neurological deficits, extent of arterial occlusion, distal perfusion, and collateral circulation. Both intra-arterial thrombolytic therapy and surgical thrombectomy have been reported to be effective in the treatment of PMA thrombosis. Thrombus resolution with restoration of PMA flow has been documented following anticoagulation. In contrast, surgical decompression or excision of the thrombosed segment may be considered in patients with severe, progressive, or refractory neurological symptoms. Because the PMA may contribute substantially to the arterial supply of the hand in some individuals, its distal connections and collateral circulation should be carefully evaluated before ligation or excision.

When vascular anatomy or distal perfusion remains uncertain, angiographic evaluation may further delineate the palmar and digital arterial circulation and facilitate treatment planning. Management should also address concomitant median nerve compression and potential mechanical aggravating factors. Depending on symptom severity, temporary neutral-position wrist splinting, modification of repetitive wrist-loading activities, and avoidance of further local mechanical stimulation may be considered. Serial neurological examination is important for detecting progression of sensory or motor deficits, while follow-up gray-scale and Doppler ultrasonography can provide a noninvasive means of monitoring thrombus evolution, arterial recanalization, median nerve morphology, and distal perfusion. These conservative and surveillance strategies should complement anticoagulation or invasive vascular treatment when clinically indicated.

## Conclusions

PMA thrombosis is a rare but important cause of acute or rapidly progressive median neuropathy, particularly in patients with anatomical variants such as a bifid median nerve. High-resolution grayscale and Doppler ultrasonography enable simultaneous assessment of neural involvement, arterial thrombosis, and distal perfusion. Early recognition facilitates individualized management and may help prevent irreversible neurological or ischemic complications.
